# Use of cigarettes and heated tobacco products during pregnancy and maternal–fetal outcomes: a retrospective, monocentric study

**DOI:** 10.1007/s00404-023-07101-w

**Published:** 2023-06-21

**Authors:** Giosuè Giordano Incognito, Laura Grassi, Marco Palumbo

**Affiliations:** Department of General Surgery and Medical Surgical Specialties, University of Catania, Via Santa Sofia 78, 95125 Catania, Italy

**Keywords:** Pregnancy, Heated tobacco products, Cigarettes, Maternal outcomes, Neonatal outcomes

## Abstract

**Purpose:**

To compare the effects of using heated tobacco products (HTP) or traditional cigarettes (C) on maternal and neonatal outcomes.

**Methods:**

This is a retrospective, monocentric study conducted at San Marco Hospital from July 2021 to July 2022. We compared a cohort of pregnant patients who smoked HTP (HS), with pregnant women smoking cigarettes (CS), ex-smoker (ES) and non-smoker (NS) pregnant women. Biochemistry, ultrasound, and neonatal evaluations were performed.

**Results:**

In total, 642 women were enrolled, of which 270 were NS, 114 were ES, 120 were CS, and 138 were HS. CS had the greatest weight gain and had more difficulty getting pregnant. Smokers and ES experienced more frequently threats of preterm labor, miscarriages, temporary hypertensive spikes, and higher rates of cesarean sections. Preterm delivery was more associated with CS and HS groups. CS and HS had lower awareness of the risks to which the mother and the fetus are exposed. CS were more likely to be depressed and anxious. Biochemical parameters did not show significant differences between the groups. CS had the greatest difference in days between the gestational age calculated based on the last menstrual period and the one based on the actual ultrasound age. The average percentile newborn weight range of CS was lower, as well as the mean 1st minute and the 5th minute Apgar scores.

**Conclusion:**

The comparison of the data obtained between CS and HS underlines the greater danger of C. Nevertheless, we do not recommend HTP because the maternal–fetal outcomes are not superimposable to the NS outcomes.

## What does this study add to the clinical work

**Table:** 

The comparison of maternal-fetal outcomes from the use of traditional cigarettes and heated tobacco products during pregnancy highlights the greater danger of the former, especially in relation to fetal growth, infertility problems, and the risk of preterm birth.	
The use of heated tobacco is not recommended, considering that obstetric outcomes are not comparable with those of non-smokers.	

## Introduction

Traditional cigarettes (C) represent a risk factor for over 17 types of tumors (on top of which lung and airway cancers) respiratory diseases (such as chronic obstructive pulmonary disease), and cardiovascular events multiplying the risk up to 5 times (as it acts damaging the endothelium and promoting atherosclerosis); it also represents a risk factor for osteoporosis, anxiety, and depression [1–4]. It can act on the reproductive system of both sexes causing a reduction in fertility [5–14], and in pregnancy, it has been shown to increase the risk of abortion [15, 16], preterm birth, premature rupture of membranes, and low birth weight [17, 18]. Despite the available data, up to 13% of pregnant women continue to smoke during pregnancy. Heated tobacco products (HTP) consist of devices placed on the market in 2014 that allow smoking of heated and unburned tobacco, thanks to the presence of a temperature regulation software that heats the tobacco contained in the stick up to 350 °C without ever reaching combustion: this would mean that about 90% of the normally toxic substances emitted by combustion are not produced, thus creating an advantage for both smokers and those exposed to smoke. It also contains about 50% less nicotine than cigarettes on the market. The reason that led us to address the present issue lies in the evidence of the growing number of smokers despite the few data in the literature, with the hope of finding data that allow us to understand if the use of HTP is less harmful than that of C. The long-term effects of these products are not yet known because of the few data in the literature despite the growing number of smokers: according to some studies, these devices could still cause inflammatory damage by increasing oxidative stress [19], while it has been reported that the effects of vasoconstriction, endothelial damage, and inflammation could be much lower than those induced by smoking [1].

## Materials and methods

### Study design and population

This is a retrospective, monocentric cohort study including pregnant women attending San Marco Hospital from 1st July 2021 to 1st July 2022. Female patients with physiologic pregnancies were included in the study. Moreover, patients with a previous or current history of obstetrics pathologies (such as gestational hypertension and/or gestational diabetes) under control as well as those with physiologic twin pregnancies were also included in the analysis. Conversely, women with previous or obstetric pathologies that could have altered the significance of the results, monochorial monoamniotic twin pregnancies, and multiple pregnancies were excluded due to their high risk of complications. Women under 18 years were excluded. The patients included were divided into the following categories: non-smokers (NS), ex-smokers (ES), traditional cigarette smokers (CS), and heated tobacco cigarette smokers (HS). It was observed that in all cases, the HTP corresponded to the same type of device, namely “IQOS”.

### Epidemiological and anamnestic evaluation

All patients underwent epidemiological evaluation through oral questionnaires and data about their age, profession, marital status, and schooling were obtained. They were all subjected to a specific smoking questionnaire, which included questions about the presence of cohabiting smokers, awareness of the risks and impact of smoking on the health of the fetus and mother, and their opinion on which product they exposed to a higher risk among CS and HS. Two questionnaires were then addressed separately which assessed the level of anxiety according to the Zung scale and the level of depression according to the PHQ-9 test. Questions about changes in smoking habits since the discovery of pregnancy and, if there was a reduction, the kind of reduction there was and in which week of gestation were asked only to smokers.

Questions about the number of cigarettes smoked daily and the duration of the smoking habit in annual terms were asked to smokers and ex-smokers. Moreover, it was asked whether smoking represented a practice sometimes aimed at replacing or reducing food consumption and appetite. The anamnestic evaluation used both oral questionnaires and patients' medical records to evaluate the number of previous pregnancies and the types of deliveries, the pre-pregnancy and the pre-partum body mass index (BMI), any difficulty in getting pregnant, any threat of preterm delivery, any blood pressure peaks during pregnancy, the gestational weeks at birth, the type of delivery, and whether labor induction was made. Finally, we recorded if a I trimester screening was done, and which test was performed between combined test and non-invasive prenatal testing (NIPT).

### Biochemical evaluation

Pre-partum biochemical data were carried out during the hospitalization and processed by the analysis laboratory located in our institution and obtained using the “Modulab” laboratory management system. These parameters included: hemoglobin (Hb) (normal value, n.v. = 11.7–65–16 g/dL), platelets (PLT) (n.v. = 150,000–450,000/mm^3^), white blood cells (WBC) (n.v. = 5200–12.400/mm^3^), prothrombin time (PT) (n.v. = 11–13.5 s), international normalized ratio (INR) (n.v. = 0.8–1.2 s), activated partial thromboplastin time (APTT) (n.v. = 26–40 s), fibrinogen (n.v. = 170–450 mg/dL), antithrombin (AT) (n.v. = 75–125%).

### Ultrasound evaluation

I, II, and III trimesters were done using the Voluson E6 ultrasound (US) system. I trimester US was performed between the 11th and the 13th gestational week, measuring the crown-rump length (CRL). II trimester morphology US was done between the 19th and 23rd gestational week, evaluating the parameters used for fetal biometry: biparietal diameter (BPD), head circumference (CC), abdominal circumference (CA), and femur length (FL). From these parameters, it was possible to calculate the gestational age (GA) based on the actual ultrasound age (AUA) according to Hedlock, and then extrapolate any difference in days with the GA calculated according to the last menstrual period (LMP), the estimated weight according to Hedlock and the corresponding weight percentile correspondent through the Intergrowth-21st software. The same procedure was applied for the evaluation of the III trimester growth US, carried out between the 29th and 33rd gestational week.

### Neonatal evaluation

Through the maternal medical records, the neonatal weight was extrapolated (from which the percentile with respect to the gestational period at the time of delivery was obtained), the 1st minute and 5th minuteApgar scores, and umbilical cord blood gas values were obtained, in particular pH (n.v. 7.35–7.45), lactates (n.v. < 4 mEq/L), and base excess (n.v. − 2/ + 2 mmol/L).

### Statistical analysis

Statistical analysis was performed by the arithmetic mean for each group of the following parameters: age, number of pregnancies, pre-pregnancy weight, pre-partum weight, pre-pregnancy BMI, pre-partum BMI, difference between pre-partum BMI and pre-pregnancy BMI, biochemical values, GA (LMP), GA (AUA), estimated weight and percentile corresponding to the I, II, and III trimester US, and the relative difference between GA (LMP) and GA (AUA), GA at delivery, neonatal weight and corresponding percentile, Apgar score, and blood gas analysis values. The percentages for each group of the following parameters were calculated: profession, marital status, education, parity, difficulty in becoming pregnant, threat of preterm birth, patients with blood pressure spikes, I trimester screening method, full-term and preterm births, type of delivery, induction and data extrapolated from smoking questionnaires.

## Results

### Epidemiological and anamnestic evaluation

In total, 642 pregnant women were enrolled, of which 270 were NS, 114 were ES, 120 were CS, and 138 were HS.

HS had the lowest mean age. Compared to the other three groups, NS more frequently had employment, were married and graduated. CS were more frequently pluriparous, had a greater weight gain (together with the HS), and had more difficulty getting pregnant. Most of the NIPTs were performed by NS and HS groups; conversely, most of the cases in which no I trimester screening test was done involved the other two groups. Smokers and ES experienced threats of preterm labor, miscarriages, temporary hypertensive spikes during pregnancy more frequently, and higher rates of cesarean deliveries (CD) than NS. Preterm delivery and induction of labor were more associated with CS and HS groups. Smokers and ES were more likely to have cohabiting smokers. CS and HS had lower awareness of the risks to which the mother and the fetus are exposed. We investigated their opinion about the possible different dangers between cigarettes and HTP, and it appeared that 55% of NS, 61% of ES, 35% of CS, and 24% of HS considered the two products at the same level of danger; on the other hand, 43%, 27%, 30%, and 76% of the respective categories believed that cigarettes expose to more risks, and 2%, 12%, 35%, and 0%, respectively, believed that HTP were more dangerous for maternal and fetal health. CS were more likely to be depressed according to the PHQ-9 test and anxious according to Zung than the other groups. 60% of CS and 78% of HS reduced the daily number of cigarettes smoked, and this occurred on average at 5 gestational weeks of amenorrhea. CS on average smoked more cigarettes per day and for more years than HS and ES. Finally, smokers and ES did not present significant differences on whether smoking constituted an expedient to replace and reduce the consumption of food. Epidemiological and anamnestic characteristics are illustrated in Table 1.

**Table 1:** Epidemiological and anamnestic evaluation

	Non-smokers	C smokers	HTP smokers	Ex-smokers
Number	270	120	138	114
Age (years)	30	30.5	27.9	30.8
Profession	35% employed 58% unemployed 7% students	15% employed 80% unemployed 5% students	35% employed 65% unemployed	38% employed 55% unemployed 7% students
Marital status	68% married	40% married	52% married	55% married
School attendance	33% degree 47% high school 20% middle school	23% degree 47% high school 30% middle school	25% degree 40% high school 35% middle school	22% degree 50% high school 28% middle school
Cohabiting smokers	13%	85%	65%	55%
Awareness of smoking risks	100%	65%	78%	94%
Comparison between C and HTP	55% think they are the same 43% think C are worse 2% think HTP are worse	35% think they are the same 30% think C are worse 35% think HTP are worse	24% think they are the same 76% think C are worse 0% think HTP are worse	61% think they are the same 27% think C are worse 12% think HTP are worse
PHQ-9 depression scale	80% subthreshold 9% low 11% moderate	75% subthreshold 25% moderate	82% subthreshold 13% low 5% moderate	85% subthreshold 10% low 5% moderate
Zung anxiety scale	85% very low 7% low 7% moderate	30% very low 25% low 30% moderate 15% high	40% very low 25% low 23% moderate 12% high	55% very low 33% low 6% moderate 6% high
Reduce of smoking habits in pregnancy		60%	78%	
Cigarettes per day (*n*)		12	8	8
Smoke duration (years)		13	5	10
Smoke in replacement of food		35%	34%	30%
Pregnancies (*n*)	2.06	2.75	1.9	2.1
Pre-pregnancy weight (Kg)	68.7	63.7	60.6	62.7
Pre-partum weight (Kg)	81.5	79.5	75.3	75
Pre-pregnancy BMI (Kg/m^2^)	25.3	23.4	23	22.4
Pre-partum BMI (Kg/m^2^)	30.2	29.45	28.8	27.6
Pre-partum BMI–pre-pregnancy BMI (Kg/m^2^)	4.9	6	5.8	5.2
Infertility	8%	25%	9%	7%
Threat of preterm birth	4%	20%	17%	11%
Gestational week at delivery	38 + 6	38 + 3	39 + 1	39 + 4
Preterm birth	4%	20%	17%	11%
Type of birth	40% CD 60% VD	51% CD 49% VD	52% CD 48% VD	55% CD 45% VD
Birth induction	24%	50%	54%	35%
Prenatal screening	70% combined test 19% NIPT 11% no screening	70% combined test 7% NIPT 23% no screening	74% combined test 18% NIPT 8% no screening	66% combined test 13% NIPT 21% no screening

*BMI* body mass index,* C* traditional cigarettes,* CD* cesarean delivery,* HTP* heated tobacco products, *n* number,* NIPT* non-invasive prenatal test,* VD* vaginal delivery

### Biochemical evaluation

Biochemical parameters did not show significant differences between the groups, although CS had slightly lower Hb and higher PLT and WBC values. Biochemical data are displayed in Table 2.

**Table 2:** Biochemical evaluation

	Non-smokers	C smokers	HTP smokers	Ex-smokers
Hemoglobin (g/dl)	11.3	10.81	11.1	11.1
Platelets (n/mm^3^)	211.300	256.000	218.000	238.166
White blood cells (n/mm^3^)	12.124	13.552	12.362	12.288
PT (sec)	10.82	10.93	10.79	10.95
INR (sec)	0.96	0.96	0.98	1.02
APTT (sec)	27.9	28.2	28	28
Fibrinogen (mg/dl)	415	496.5	461	422
Antithrombin (%)	91.3	90.8	90.6	90

*APTT* activated partial thromboplastin time,* C* traditional cigarettes,* HTP* heated tobacco products,* INR* international normalized ratio,* PT* prothrombin time

### US evaluation

In the II trimester US, CS had the greatest difference in days between GA (LMP) and GA (AUA) (NS = 0 vs. CS = 4 vs. HS = 1 vs. ES = 2), with greater involvement of the AC (NS = 152.2 vs. CS = 139.5 vs. HS = 156.9 vs. ES = 150.3), and the lowest weight percentile (NS = 60 vs. CS = 16 vs. HS = 58 vs. ES = 47).

Regarding the III trimester US, CS had the greatest difference in days between GA (LMP) and GA (AUA), followed by ES (NS = 0 vs. CS = 7 vs. HS = 0 vs. ES = 4), with greater involvement of the AC (NS = 276.0 vs. CS = 250.7 vs. HS = 270.3 vs. ES = 270.3). CS and ES had the lowest weight percentile (NS = 62 vs CS = 34 vs HS = 70 vs ES = 30). US outcomes are reported in Table 3.

**Table 3:** Ultrasound evaluation

	Non-smokers	C smokers	HTP smokers	Ex-smokers
I trimester US				
GA (LMP)	12 + 4	12 + 4	12 + 3	12 + 2
CRL	59.1	58.3	58.1	59.4
II trimester US				
GA (LMP) (weeks + days)	20 + 6	21 + 0	20 + 6	20 + 6
GA (AUA) (weeks + days)	20 + 6	20 + 1	21 + 0	20 + 4
GA (LMP)—GA (AUA) (days)	0	4	1	2
BPD (mm)	52.2	50	51.9	50.4
CC (mm)	181.2	183.7	184.5	182.1
CA (mm)	152.2	139.5	156.9	150.3
FL (mm)	33.7	32.4	36.13	33.3
Estimated weight (gr)	357	309.4	351.5	323.9
Percentile (°)	60	16	58	47
III trimester US				
GA (LMP) (weeks + days)	31 + 4	31 + 4	31 + 0	32 + 3
GA (AUA) (weeks + days)	31 + 4	30 + 6	31 + 0	31 + 5
GA (LMP)—GA (AUA) (days)	0	7	0	4
DBP (mm)	81.6	79.5	80.4	80.7
CC (mm)	294.1	288.35	291.7	292.5
CA (mm)	276.0	250.7	270.3	270.3
FL (mm)	60.5	55.1	58.43	58.2
Estimated weight (gr)	1787	1360	1646	1652
Percentile (°)	62	34	70	30

*AUA* actual ultrasound age,* BPD* biparietal diameter,* C* traditional cigarettes,* CA* abdominal circumference,* CC* cranial circumferences,* CRL* crown-rump length,* FL* femur length,* GA* gestational age,* HTP* heated tobacco products,* LMP* last menstrual period,* US* ultrasound

### Neonatal evaluation

The average percentile newborn weight range of CS was lower than the other categories of patients, as well as the mean 1st minute and the 5th minute Apgar scores. The umbilical cord gas values analyzed fell within the normal range: the only exception was found in average newborns’ pH from CS which was slightly highest. Neonatal data are illustrated in Table 4.

**Table 4:** Neonatal evaluation

	Non-smokers	C smokers	HTP smokers	Ex-smokers
Weight (gr)	3218	2890	3213	2998
Percentile (°)	50–75	10–25	25–50	25–50
1st minute Apgar score	8.8	8.4	8.8	8.7
5th minute Apgar score	9.8	9.4	9.8	9.7
pH	7.3	7.2	7.3	7.3
Lactates (mmol/L)	3.58	3.17	3.99	4.86
Bases excess (mEq/L)	− 4.47	− 3.82	− 4.98	− 6.41

*C* traditional cigarettes,* HTP* heated tobacco products

## Discussion

In this study, we analyzed systematically for the first time epidemiological, biochemical, and US data to compare the maternal–fetal outcomes between the use of C and HTP during pregnancy.

### Epidemiological and anamnestic evaluation

The epidemiological questionnaire allowed us to highlight the fact that CS were more often unemployed, single, and, together with HS, with a lower average education than NS. HS had a lower average age than the other groups, probably due to the greater diffusion of these products among the youngest. From the questionnaires relating to smoking habits, it emerged that the percentage of cohabiting smokers was much higher for CS (85%) than for other categories and that awareness of the risks to which smoking exposes was present in all NS and in almost all ES, while it was lower in CS and HS, respectively. Furthermore, their opinion on the different dangerousness between C and HTP highlighted that while most NS and ES considered HTP less dangerous or equal to the danger of C, 76% of HS considered C more dangerous, differently from CS. HS smoked on average the same amount as CS, and in both cases, more than half reduced their consumption as soon as they became aware of their pregnancy. Previous papers reported a higher rate of anxiety and depression among smokers [1–4] and these data were also confirmed by the present study. Moreover, in the present study, ES showed higher levels of anxiety than controls and this could have contributed to the cessation of smoking in anticipation of pregnancy. It has been shown that 1/3 of smokers used smoking as an expedient to replace and, therefore, reduce their food consumption. In fact, BMI gain during pregnancy was slightly higher in women who smoked. Smoking affects fertility, in fact CS in our population had infertility problems three times more often than NS, as other studies confirmed [5–14]. This occurs for several reasons: first, nicotine-induced vasoconstriction can reduce uterine blood flow and, therefore, cause difficulties in embryo implantation, and second it can interfere with estrogen production and, therefore, with menstrual cyclicality, as well as alter the process of maturation of the oocyte, damaging it and, therefore, reducing the ovarian reserve. On the other hand, HS, NS, and ES had the same rates of difficulty to get pregnant. In accordance with the literature, CS had a higher rate of miscarriages (six times higher) [15, 16] compared to NS. This correlation has been noted by several studies, even though it has never been close enough to be considered certain. To a lesser extent, HS have also been shown to have a higher abortion rate than NS, with percentages equal to those of ES. Most of the NIPT was performed by NS and HS groups, probably for a better economic condition; conversely, most of the cases in which no I trimester screening test was performed involved the other two groups. Cigarette smoking causes pro-inflammatory changes and an increase in oxidative stress, leading to various consequences: an increase in the production of prostaglandins, and therefore a stimulation of the uterine smooth muscle, an increase in the sensitivity of oxytocin receptors, and a reduction of progesterone production [17, 18]. All these changes greatly increase the risk of preterm birth. In fact, CS suffered five times more often than NS threatened with preterm birth, and they went into preterm birth four times more often. On the other hand, HS suffered of threat of preterm birth four times greater than NS, and of preterm birth two times greater than NS (in any case lower than CS). It is known that nicotine, being a vasoconstrictor agent, can induce hypertension, so cigarette smoking represents a risk factor for the development of gestational hypertension [20]. In 25% of CS, there were occasional pressure peaks during pregnancy, many more than those that occurred in other categories. Smokers of both categories and ES delivered by CD and have been subjected to labor induction more often than NS.

### Biochemical evaluation

The blood count values were comparable among the categories of NS, ES, and HS, while they reveal slight alterations in CS, in particular: lower Hb values, and higher PLT and WBC values. The slightly lower Hb value could be explained by the fact that the carbon monoxide contained in tobacco interferes with the transportation of oxygen in the blood [21], while the higher WBC values could be due to the induced pro-inflammatory state from smoking. Values of the coagulation tests were comparable in the four categories, except the fibrinogen which was on average higher in CS, and these could be influenced by tobacco, representing a risk factor especially for venous thrombosis.

### US evaluation

In the morphological US performed on CS, some data of fetal biometry were lower than in other categories, in particular those regarding the AC, as already stated in several studies [22]. GA (AUA) was on average lower than 4 days compared to GA (LMP), as well as the estimated weight (when compared to the corresponding percentile) was lower. In the growth US, some values of fetal biometry were lower in CS than in other categories too, as in the II trimester US. GA (AUA) was lower than 7 days compared to GA (LMP), as well as the estimated weight in relation to the growth percentile. In fact, several studies stated that the impact of smoking on fetal growth is visible since nutritional requirements increase, therefore from the II trimester onwards [23]. Compared to the morphological US values, the differences are more evident in the growth US, that is, in fact the period in which the uterus-placental flow must satisfy a greater nutritional requirement, precisely because the fetus has reached a higher weight. The fetuses of ES also showed a reduction in growth which becomes evident in the III trimester, and this testifies to the persistence of the vasoconstrictive effects induced by smoking that could become evident when fetal nutritional requirements increase. In fact, nicotine, as well as other toxic substances present in smoke, induces vasoconstriction which, as stated by several studies [24], can reduce the blood supply to the fetus, and therefore cause type 2 intrauterine growth restriction.

### Neonatal evaluation

As other studies supported [25], the average newborn weight from CS was lower than the other categories, if compared with the corresponding percentiles: in particular, it was between the 10th and 25th percentiles (while in NS was between the 50th and 75th percentiles). On the other hand, newborns from HS had a slightly lower weight when compared to those born from NS (between the 25th and 50th percentiles), but were still similar to those of ES. The 1st minute and 5th minute Apgar scores from CS were slightly lower than the other groups. Dawood et al. [26] found that in heavy smokers, neonatal Apgar can have values even four times lower than the average. The umbilical cord gas values show physiological changes in the term newborn, such as a lower pH (7.18–7.38), and a base excess with lower values (between − 8 and − 0 mEq/L). All values analyzed in the present study were within the normal range: the only exception was found in those born from CS, in which an average pH value was slightly higher than in the other categories. As reported in previous studies [27], this could be linked to maternal hyperventilation caused by cigarette smoke which could also be reflected in the fetal acid–base balance. In fact, in this category, the lactate values were lower, and the base excess values were higher, in accordance with the pH. Due to the lack of long follow-up, we could not evaluate some newborn parameters, such as lung function during development, which was, however, evaluated in other studies in which reduced lung function was found in children born from mothers who smoked during pregnancy, as well as increased risk of developing asthma and lung infections [28, 29]. Moreover, it was not possible to evaluate the development of other organs, although several studies found, in some cases, underdevelopment of some organs (such as liver and kidneys) in children born from CS [30, 31]. The reason why maternal and fetal outcomes have a lower impact in HS than in CS could be explained by the fact that the amount of nicotine is 85% lower in HTP compared to the average values found in C, as well as the absence of numerous toxic substances released by C combustion (primarily carbon monoxide) which contribute to cause many obstetric problems. The values examined in ES showed that the effects of tobacco on the body can persist in the long term even after years of cessation. In fact, the percentages of miscarriages, preterm births, and blood pressure peaks were higher when compared with the percentages of NS. The II and III trimester fetal growth values observed by US were also lower than those found in NS.

This study has several strengths: it is the first of its kind to assess the risks associated with the use of HTP in pregnancy, the fact that the women were hospitalized and allowed us to carry out a precise anamnestic evaluation; the biochemical and US assessments were conducted at a single center. Nevertheless, it has some limitations: the design of the study (retrospective), and the total number of patients evaluated. Moreover, a large confounder is the fact that women who smoked appear to be older and from lower socio-economic levels than those who used heated tobacco products. Women who used heated tobacco products also appeared to be using it for fewer years than smokers. This potential bias may have influenced the results on the risk of preterm birth, infertility, and fetal growth restriction. Therefore, although there is a suggestion that heated tobacco products cause less harm, the risk of bias is present, and we cannot make a firm conclusion that using heated tobacco products is safer than smoking.

In conclusion, the comparison of the data obtained between CS and HS underlines the greater danger of C compared to HTP, especially in relation to fetal growth evaluated in different US, infertility problems, and the risk of preterm birth. The comparison between values obtained in NS and ES with those obtained in HS did not reveal any noteworthy differences, either at the anamnestic, laboratory or US level. A slight increase in the percentage of infertility, miscarriages, and preterm births was found in HS compared to NS, but comparable with the percentages found in ES. In fact, compared to NS, ES had a higher rate of miscarriages, preterm births, and blood pressure peaks, as well as a slight reduction in II and III trimester fetal growth parameters. Some effects induced by nicotine and tobacco toxicants are likely to persist long-term even after smoking cessation. These results do not represent an encouragement to the use of heated tobacco, which, therefore, is not recommended, considering that obstetric outcomes are not comparable with those of NS. Furthermore, more numerous and prospective studies, with long-term follow-up, are necessary to confirm these data and clarify the exact degree of danger associated with the use of HTP during pregnancy.
